# Teaching family medicine: The National Postgraduate Medical Curriculum Training the Trainers over the years

**DOI:** 10.51866/mol.1154

**Published:** 2026-06-15

**Authors:** Farnaza Ariffin

**Affiliations:** 1 Department of Primary Care Medicine, Faculty of Medicine UiTM, Sungai Buloh Campus, Sungai Buloh, Selangor, Malaysia.

**Keywords:** NPMC, Training the trainers, Course directors, Family medicine, Curriculum

## Training needs

Doctors are, by definition, teachers – a fact reflected in the Latin origin of the title, *docere.* However, effective teaching requires more than just clinical knowledge; it requires specialised training. This is where the National Postgraduate Medical Curriculum (NPMC) Training the Trainers (TtT) initiative becomes essential. The family medicine fraternity played a pivotal role during the development of the NPMC project. This ambitious project aims to deliver a unified and structured curriculum for the training of specialists throughout Malaysia. To ensure this curriculum is delivered effectively, the TtT steering committee developed a specialised module to enhance the expertise of clinical trainers. Through a network of appointed course directors from each discipline, the programme now facilitates standardised, high-quality instruction across the specialty training.

## The experience

I have been fortunate enough to be part of the NPMC project since its beginning. My journey started in 2016 as a member of the curriculum writing group, which eventually led to the proud moment in 2021 when we launched *The Primary Care Medicine Postgraduate Training in Malaysia curriculum* book. I was also honoured to be appointed as the first course director for family medicine. This allowed me to transition into a new role: leading the introduction and delivery of the TtT workshops for our fraternity. In 2024, I was appointed as a TtT steering committee member.

## The progress

**Figure 1:**
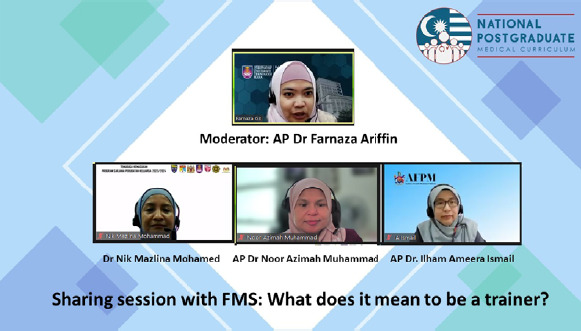
The inaugural NPMC Training the Trainers in Family Medicine in 2022 was conducted online.

In 2022, we reached a major milestone with the launch of our first online NPMC TtT course in family medicine (Figure 1). The overwhelming response from our inaugural participants sparked momentum that has grown, leading us to establish the course as a highly anticipated annual event. Over the past 5 years, we have successfully delivered five courses through diverse platforms – online, in-person and hybrid. We have successfully trained a total of 205 dedicated educators. Our leadership has also expanded significantly, and we now have 15 course directors representing the Peninsular states and East Malaysia. It is inspiring to see this event bring together trainers from all three postgraduate tracks - the Master’s, AFPM and MInTFM programmes. By unifying these pathways under a single standard of excellence and with the AFPM now incorporating the NPMC into their annual workshops, we are ensuring a stronger, more cohesive future for primary care training in Malaysia.

**Figure 2:**
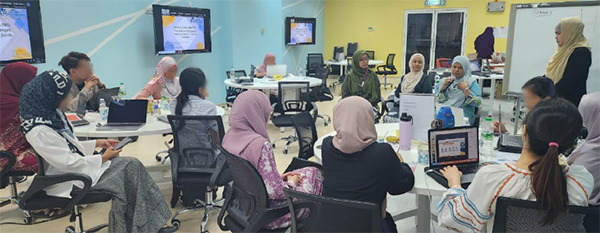
The synchronous session is packed with interactive lectures and group work skills activities to ensure engagement and understanding of key concepts within the module.

Built on a structured NPMC model, our course combines asynchronous video modules and assessments with a dynamic, 2-day interactive workshop (Figure 2). These sessions deliver key concepts on clinical reasoning, feedback and reflective practice. We also empower trainers with leadership and supervision skills, providing a safe space to discuss training challenges and strategies for managing oppressive behaviours.

## The future

Our vision for the future is to improve regional access to the TtT course through smaller workshops led by local course directors, especially in the East Coast and East Malaysia. Accordingly, course directors will be trained to achieve mastery of the NPMC modules and deliver core concepts with precision. To maintain a high standard, we aim to re-train all trainers every 5 years.

